# *Mucorales* and Mucormycosis: Recent Insights and Future Prospects

**DOI:** 10.3390/jof9030335

**Published:** 2023-03-09

**Authors:** Ghizlane Tahiri, Carlos Lax, José Tomás Cánovas-Márquez, Pablo Carrillo-Marín, Marta Sanchis, Eusebio Navarro, Victoriano Garre, Francisco Esteban Nicolás

**Affiliations:** 1 Departamento de Genética y Microbiología, Facultad de Biología, Universidad de Murcia, 30100 Murcia, Spain; 2 Serra Húnter Fellow, Facultad de Medicina y Ciencias de la Salud, Departamento de Ciencias Médicas Básicas, Unidad de Microbiología y Microbiología Ambiental, Universidad Rovira i Virgili, 43003 Reus, Spain; 3 Institut d’Investigació Sanitària Pere Virgili (IISPV), 43003 Reus, Spain

**Keywords:** *Mucorales*, mucormycosis, antifungal resistance, *Mucor*, *Rhizopus*

## Abstract

The classification of *Mucorales* encompasses a collection of basal fungi that have traditionally demonstrated an aversion to modern genetic manipulation techniques. This aversion led to a scarcity of knowledge regarding their biology compared to other fungal groups. However, the emergence of mucormycosis, a fungal disease caused by *Mucorales*, has attracted the attention of the clinical field, mainly because available therapies are ineffective for decreasing the fatal outcome associated with the disease. This revitalized curiosity about *Mucorales* and mucormycosis, also encouraged by the recent COVID-19 pandemic, has spurred a significant and productive effort to uncover their mysteries in recent years. Here, we elaborate on the most remarkable breakthroughs related to the recently discovered genetic advances in *Mucorales* and mucormycosis. The utilization of a few genetic study models has enabled the identification of virulence factors in *Mucorales* that were previously described in other pathogens. More notably, recent investigations have identified novel genes and mechanisms controlling the pathogenic potential of *Mucorales* and their interactions with the host, providing fresh avenues to devise new strategies against mucormycosis. Finally, new study models are allowing virulence studies that were previously hampered in *Mucorales*, predicting a prolific future for the field.

## 1. Introduction

The progression of microorganisms embodies a persistent emergence of pathogens that affect humans, including new variants of bacteria and fungi that elude existing antibiotics and antifungal treatments. Among the fungi, the *Mucorales* order is a wellspring of highly resistant species responsible for mucormycosis, a deadly and emerging infection [1]. This disease is the third-most-common angio-invasive fungal infection, following candidiasis and aspergillosis, in patients with hematological and allogeneic stem cell transplantation [2]. With an aging population, the increased number of immunocompromised patients, and the recent COVID-19 pandemic, the number of individuals susceptible to Mucorales infections is on the rise [3]. The noticeable increase in mucormycosis cases, a mortality rate of 90% for disseminated infections, and the absence of effective antifungal treatments have triggered widespread concern regarding this emerging disease [4,5]. In addition, advancements in diagnostic techniques have uncovered an alarming number of cases of mucormycosis among immunocompetent/otherwise healthy individuals [6]. The *Mucorales* are often overlooked compared with other fungi, such as Ascomycetes and Basidiomycetes. The limited understanding of the genetics of *Mucorales* is due to their resistance to modern genetic manipulation techniques, as many cannot be transformed. However, the persistence of the scientific community has found a few species, such as *Mucor lusitanicus* (previously known as *Mucor circinelloides f. lusitanicus*) and *Rhizopus microsporus,* which are opening this field to genetic manipulation [7,8,9]. The increasing concern about emerging cases of mucormycosis, coupled with these genetic models, has sparked interest among the scientific community. As a result, the past decade has seen a surge in studies on genes, pathways, and mechanisms that directly correlate with virulence and antifungal resistance in *Mucorales*. One of the most thoroughly examined mechanisms has been the process of gene silencing or RNA interference (RNAi) in *M. lusitanicus*. Following the dissection of the gene-silencing machinery, knowledge of this mechanism led to the discovery of a novel and specific type of antifungal resistance mediated by temporal epigenetic changes [10]. The application of gene silencing as a genetic tool has facilitated the development of functional genomic techniques, which have been used to identify several new virulence factors. Along with silencing, gene disruption through homologous recombination has also enabled the study of the specific role of virulence factors in *M. lusitanicus*, identified in other fungi, such as the role of a high-affinity iron-uptake mechanism, the CotH protein family, and the calcineurin pathway. Furthermore, the advent of new omics technologies has produced a long list of candidate genes not previously linked to virulence, providing promising targets for developing new treatments for mucormycosis. Finally, the diversity of molecular and cellular methodologies has allowed for the study of the genetic response during host–pathogen interactions, revealing the crucial role of several regulatory genes.

## 2. Genes Involved in the Pathogenic Potential of *Mucorales*

### 2.1. The High-Affinity Iron Uptake System in Mucorales

Iron is an essential micronutrient for the different organisms across the life kingdoms. Due to its fundamental functions in the physiology of living organisms, it has a substantial role in maintaining the virulence of pathogens. Conversely, hosts have evolved to hide their iron reservoirs from pathogens to diminish their virulence [11]. In the case of *Mucorales* and mucormycosis, several studies established an apparent increase in virulence when the host iron-hiding systems fail, and an abnormally high free iron level is observed in blood [12]. Moreover, *Mucorales* have developed their own systems to acquire iron from their hosts, highlighting the vital role of iron for these pathogens during infection [13,14,15].

One of these systems is the high-affinity iron-uptake mechanism, a three-component-based mechanism depending on iron reduction, oxidation, and incorporation activities. These enzymatic activities are performed by the iron reductase Fre, the ferroxidase Fet3, and the permease Ftr1 [15,16,17]. A failure in the high-affinity iron-uptake system of *Mucorales* inevitably leads to a decrease in their virulence [16,18] These failures, induced by directed mutagenesis, were mainly studied in the genetic model *M. lusitanicus* [15]. This fungus has three paralogous ferroxidases genes, fet3a, fet3b, and fet3c, with fet3c being the key virulent factor, although a partial redundancy exists with the other two paralogs. However, the most intriguing result from this genetic analysis was the sub-functionalized role of the three paralogs during dimorphism. The genes fet3b and fet3c are highly expressed in the yeast form, whereas fet3a is only expressed in the mycelium [15]. Only the mycelial form is capable of developing mucormycosis, which settles the process of dimorphism as another determinant involved in virulence (elaborated below). The differential expression of the three fet3 genes in the two dimorphic states of *M. lusitanicus* was the first evidence connecting the high-affinity iron-uptake mechanism and dimorphism, linking two crucial processes involved in the virulence of *Mucorales*. Besides the role of iron ferroxidases in *M. lusitanicus*, the iron permease Ftr1 was studied in Rhizopus delemar, showing that a reduced expression correlated with decreased virulence [16] (Table 1).

Another system developed by pathogens to acquire iron from the host is based on small compounds with high-affinity iron-chelating activity known as siderophores. In the case of *Mucorales*, the most specific siderophore is rhizoferrin, but this is also the least studied in mucormycosis [13,14,31]. Fungi synthesize their own siderophores but can also use the siderophores produced by other microorganisms [32]. Moreover, Rhizopus spp. can use the synthetic siderophore deferoxamine, a siderophore used in dialysis patients with iron overload [33].

### 2.2. Azole Resistance in Mucorales: The Cytochrome P450

Azoles are a group of antifungal compounds commonly used in the clinic against fungal infections. Among them, those with three nitrogen atoms and a cyclic structure are known as triazoles. Their antifungal mechanism relies on suppressing the cytochrome P450 activity, which is mainly involved in synthesizing ergosterol [34,35,36]. Azole resistance spontaneously appears in fungi [37] by three different mechanisms: increasing the amount of P450, decreasing its inhibition, or increasing the azole transport out of the cell [38,39]. However, *Mucorales* present an innate and general azole resistance higher than other fungi [1,40,41]. This resistance explains the lack of effective antifungal treatments against *Mucorales*, and it is the main reason forcing clinicians to continue using old compounds such as amphotericin B to treat mucormycosis infections [1,42,43]

Among the different cytochrome P450 enzymes, the lanosterol 14*α*-demethylase CYP51 (also known as Erg11) plays a critical role in azole antifungal resistance in Aspergillus [44,45,46,47,48]. Similarly, a recent study found a possible link between specific mutations in CYP51 and the innate triazole resistance observed in *Mucorales* [19,49]. *Mucorales* have two paralogues of CYP51, CYP51 F1 and CYP51 F5. The amino acid sequence analysis of different *Mucorales* showed only two conserved mutations in F5, which create a predicted structural change that might explain how short-tailed azoles cannot inhibit this version of CYP51 [49] (Table 1). However, this predicted model still requires experimental validation.

### 2.3. The cotH Gene Family, an Important Source of Virulence Factors in Mucorales

The cotH gene family encodes unconventional protein kinases that are found in spores of different organisms, both prokaryotes and eukaryotes [50,51]. They are related to the regulation of the spore integrity, and mutants affected in their sequence are frequently associated with defective germination [52]. The genomes of different *Mucorales* contain several copies of cotH genes, and the proteins are found in the spore surface [52,53]. Usually, mucoralean species lacking these proteins are avirulent, and a higher number of cotH gene copies is associated with more aggressive species [53,54]. The mechanism relating CotH proteins to virulence is associated with adherence and tissue invasion. Thus, CotH proteins have a conserved motif that interacts with the host endothelial cells, initiating the tissue invasion [53,55,56].

A recent study found 17 cotH-like genes in the genome of Mucor lusitanicus. The disruption of five of them led to defects in temperature adaptation and cell wall development. More importantly, their role in virulence was confirmed in a mouse model [20] (Table 1). Besides some bacteria and *Mucorales*, CotH proteins are not found in other fungi such as Candida and Aspergillus, making them a specific target for therapeutic and diagnostic approaches [21,57].

### 2.4. Genomic Approaches to Identify New Virulence-Related Genes

Although most of the genetic analyses described in the previous sections dissected the role of genes identified in other pathogenic fungi, the current omic technologies allow more ambitious projects to search for new virulence factors in *Mucorales*. Two approaches explored mucoralean genomes, trying to find new virulence determinants that were not previously described in other pathogenic organisms.

The first one performed a comparative genomic approach confronting the genomes of two highly related strains of *M. lusitanicus*: CBS277.49 and NRRL363 [22]. These two strains share identical features in most of their fungal physiology except for a critical aspect: their pathogenic potential. The strain CBS277.49 is virulent and kills most of the hosts in survival assays, whereas NRRL363 is avirulent and is usually chosen as a negative control. Thus, the comparison between their genomes is expected to find key differences that might be involved in the pathogenic phenotype. This approach identified 543 absent genes and 230 discontiguous protein-coding sequences in the avirulent strain [22]. The functional screening of those genetic differences identified a secreted protein with unknown functions that was highly involved in the virulence of CBS277.49 [22] (Table 1). As expected, other differences between CBS277.49 and NRRL363 strains, such as the gene ID108920 (hypothetical g-glutamyltranspeptidase), had no impact on the virulent phenotype. Another study with a different perspective also compared mucoralean genomes, but in this case, all of them were from mucormycosis-causing isolates searching for similarities. This approach revealed that a higher copy number of cotH genes correlates with strong virulence and clinical prevalence [53].

In a similar comparative approach but at the RNA level, a transcriptomic analysis comparing the avirulent NRRL3631 and the virulent strain CBS 277.49 led to the identification of Wex1, a new exonuclease involved in virulence [23]. Moreover, transcriptomic analysis during macrophage–spore interaction allowed for the identification and further genetic dissection of two Atf transcription factors and their regulated targets [24] (Table 1).

The second approach developed a methodology for identifying new virulence factors at the genomic scale. This methodology developed a functional genomic strategy using an RNAi high-throughput library that allowed for the fast screening of new virulence factors. Briefly, a collection of plasmids capable of silencing all the genes of *M. lusitanicus* enabled the isolation of transformants with interesting phenotypes. Later, a fast screening in Galleria mellonella selected only the transformants with phenotypes related to virulence. Then, the plasmids in the selected transformants were rescued and sequenced, unveiling the genes responsible for the virulence-related phenotype. Finally, deletion mutants were generated and validated in a murine survival assay. The first “proof of concept” application of this methodology identified two previously unknown virulence determinants: the genes mcplD and mcmyo5 [25] (Table 1).

## 3. Gene Pathways with Pleiotropic Effects on the Pathogenic Potential of *Mucorales*

### 3.1. Dimorphism Controls Virulence in M. lusitanicus

Dimorphism yeast/mycelium has been related to virulence in several pathogens, with different conclusions. Thus, pathogenic fungi such as *Talaromyces marneffei*, *Blastomyces dermatitidis*, *Coccidioides immitis*, *Histoplasma capsulatum*, *Paracoccidioides brasiliensis*, *and Sporothrix schenckii* turn to the yeast form when they are invading a host [58]. On the contrary, in Candida albicans, the main human fungal pathogen, both morphological forms are involved in its pathogenic potential, with the transitioning capacity being the clue for virulence [59]. In the case of *Mucorales*, dimorphism and its role in virulence have been studied in the fungus *M. lusitanicus*. In this fungus, the lack of oxygen and the presence of CO_2_ and fermentable hexoses induce yeast growth, whereas oxygen and starvation stimulate hyphal growth [60,61]. Regarding virulence, several studies pointed out that only the filamentous form of *M. lusitanicus* is involved in virulence. In these studies, the multifunctional regulator Calcineurin controlled the pathway regulating dimorphism [26,27]. Calcineurin harbors two subunits, a catalytic phosphatase (subunit A) and a regulatory subunit B. When subunit B is activated by calcium-calmodulin, the catalytic subunit dephosphorylates transcription factors, leading to changes in the expression of target genes [58]. Mutations in the Calcineurin of *M. lusitanicus* resulted in a yeast-locked form and an avirulent phenotype [26,27].

Another essential pathway controlling dimorphism in *M. lusitanicus* is the cAMP-dependent protein kinase A (PKA) [61]. PKA is a complex enzyme containing two regulatory (PKAR) and two catalytic (PKAC) subunits [28]. *M. lusitanicus* has four genes coding PKAR subunits, but only three are involved in dimorphism (PKAR1, PKAR2, and PKAR4) [28]. In addition, the heterotrimeric G-protein beta subunit 1 (Gpb1) regulates PKAR1, and mutations in their genes result in the same phenotype [29] (Table 1).

### 3.2. The Epimutant RNAi Pathway and Its Role in Antifungal Resistance

One of the unique features of *Mucorales* is their high-level resistance to most available antifungals, which explains the lack of effective treatments and the high mortality rate of mucormycosis [62]. In this sense, a research line studying the RNAi pathway focused its efforts on unveiling the link between this pathway and the antifungal resistance of *Mucorales*. Firstly, the primary RNAi mechanism of *M. lusitanicus* was genetically dissected, characterizing the main components and their functions [63]. These studies showed a foundation RNAi core in *M. lusitanicus* that is highly conserved [64,65,66]. However, as these studies deepened the mucoralean RNAi mechanism, several modifications of the RNAi central core revealed the existence of three different RNAi pathways in *M. lusitanicus*: the canonical, the non-canonical, and the epimutational pathways [10,67,68,69,70]. The canonical pathway shares mechanisms, components, and functions with common RNAi pathways found in other eukaryotes. Conversely, the non-canonical pathway is Dicer- and Argonaute-independent, performing unique functions (elaborated in the next section). The third pathway, the epimutational mechanism, is also canonical in the sense of its requirements of Dicer and Argonaute components. However, it requires other components, such as an exonuclease similar to the quelling-induced protein (QIP) and a Sad-3-like helicase (RnhA) [69]. The main particularity of the epimutational pathway is its function, which is related to the phenotypic adaptation to stressful conditions in the environment. For instance, this epimutational pathway confers antifungal resistance to the fungus and its progeny as long as the antifungal compound is present in the medium. Furthermore, the antifungal resistance is temporarily acquired, as it disappears when the antifungal compound is removed [10]. The molecular mechanism behind this antifungal resistance is based on the specific production of sRNAs targeting only the mRNA encoding the enzymes involved in antifungal drug sensitivity. Thus, the lack of the target enzymes allows the fungus to thrive in the presence of the drug. When the drug is removed from the medium, the fungus terminates the production of the specific sRNAs and restores the expression of the target gene [62,71].

The epimutational pathway has only been reported in *Mucorales*, which makes it a perfect target for the development of new antifungal drugs. In this regard, the lack of QIP and RnhA in mammal hosts could represent an opportunity to develop specific inhibitors with antifungal properties.

### 3.3. The Non-Canonical RNAi Pathway and Its Role in the Pathogenic Potential of Mucorales

The non-canonical RNAi pathway (NCRIP) is another particularity described only in *Mucorales*; therefore, it is another interesting target for developing new antifungal drugs. The NCRIP is RdRP-dependent and Dicer-independent, relying on the RNase III activity in the protein R3B2 [70,72]. The functional role of NCRIP has been related to RNA degradation, being postulated as an evolutionary link between the ancient bacterial RNA degradation mechanisms and the modern eukaryotic RNAi canonical mechanisms [69,70]. More recent studies also related the functional role of NCRIP to the maintenance of genome stability and virulence [30].

Mutants affected in the NCRIP mechanism lacked the full pathogenic potential to kill the host [30]. The role of NCRIP in the virulence of *Mucorales* was studied in the initial phase of the infection, during the interaction with the phagocytic cells of the host. In this interaction, mutant spores lacking the NCRIP mechanism showed a strong misregulation of gene expression in response to phagocytosis [30,73]. A thorough analysis of the misregulated gene in NCRIP mutants showed that the role of this mechanism is to repress a pool of genes that must be activated only during phagocytosis. Therefore, NCRIP mutants activated this pool of genes without the stimulus of phagocytosis during non-stressful conditions. The same analysis found numerous genes related to the oxidative stress response among the genes misregulated during phagocytosis [30]. Previous studies found that the NCRIP mechanism regulates hundreds of genes; therefore, a pleiotropic effect was expected in the mutants lacking this mechanism [70]. Among the observed phenotypes in NCRIP mutants, an altered response to oxidative stress was confirmed [70]. Later, those observations were connected with the pool of genes misregulated during phagocytosis, hypothesizing that this connection could explain the lack of virulence [30]. Thus, a proper response to oxidative stress could relate to a natural scenario such as the oxidative environment inside macrophages after phagocytosis. The misregulation of this response could affect the capability of these fungi to overcome the phagocytic attack [30].

Moreover, the key enzyme of this mechanism, the RNase R3B2, is also specific to the *Mucorales* group, becoming another promising target for developing new antifungal drugs. The structure and function of R3B2 have been studied in detail [74]. This special RNase possesses an RNase III-like domain (RIIID), which allows the Dicer-independency of the NCRIP mechanism. Several pieces of evidence suggest that R3B2 dimerizes by RIIID, similarly to Dicer, Drosha, and bacterial RNases III [74]. However, Dicer and Drosha dimerize to cut double-stranded RNA (dsRNA), whereas the experimental evidence shows an R3B2 preference for single-stranded RNA (ssRNA) [74,75,76,77]. Among its particularities, R3B2 has two double-stranded RNA binding domains (dsRBD), which contradicts its ssRNA degradations activity. This contradiction has been resolved with the crystallographic structure of the R3B2-RIIID. Most of the R3B2-RIIID is similar to other RNases III, explaining its dimerization ability. Nevertheless, R3B2-RIIID lacks a motif essential for dsRNA binding in canonical RNAses III: the linker α5/α6 [78]. Moreover, the R3B2-RIIID also has a narrower catalytic center than canonical RNases III, suggesting a steric impediment to the entrance of dsRNAs that would explain the preference for ssRNA [74]. All these singularities and the exclusiveness of NCRIP in *Mucorales* support the development of related antifungal drugs as new and specific treatments for mucormycosis.

The epimutational and the NCRIP mechanisms have distant functional roles and machinery. Moreover, the epimutational mechanism is triggered by environmental stress, whereas NCRIP acts during normal growth conditions [69]. These differential roles correlate with their opposite regulation in which NCRIP represses the epimutational pathway, likely by competing for the same target RNAs [69]. Consequently, mutants affected in the NCRIP mechanism exhibit an activated epimutational pathway with a higher production of epimutants.

## 4. Genetic Manipulation in *Mucorales* and New Study Models

### 4.1. In Vitro Models to Study the Host–Pathogen Interaction 

The natural route of infection by *Mucorales* is the contact of vegetative spores with the respiratory mucosa or an open skin wound. There, the first host response is the innate immune system, and the phagocytosis of the spores is the primary mechanism to avoid fungal invasion [73]. Among phagocytic cells, neutrophils and macrophages are the key effectors recruited to the infected area for the clearance and killing of the fungal spores. In this sense, an in vitro model uses the immortal cell line J774A.1, derived from mouse macrophages and conserving their morphology, adherence, and phagocytic potential. This model served to study the genetic response of both the host and the mucoralean pathogen during phagocytosis [24]. This study unveiled the genetic profile triggered in the spore of *M. lusitanicus* after phagocytosis, showing the primary and secondary genetic responses triggered by the activation of the master genes *atf-1* and *atf-2* [24] (Table 1).

Similar to the increased susceptibility to mucormycosis observed when macrophages are depleted, neutropenia is also a risk factor associated with this disease, highlighting the pivotal role of neutrophils [79,80]. Accordingly, the in vivo studies developed in zebrafish larvae showed that the formation of the primary granulomas is executed by neutrophils [81]. However, these primary granulomas have not yet been isolated or reproduced in an in vitro model, impairing the dissection of this process.

Another established in vitro model to study the host–fungal spore interaction is based on epithelial cells. These cells are the first mechanical barrier defending the host from pathogenic microorganisms. Consequently, injuries and lesions in these defensive epithelia are risk factors for mucormycosis [29]. The in vitro model uses the alveolar epithelial cell line A-549 [14,31]. The host transcriptional response during the interaction with mucoralean spores revealed the activation of signaling pathways such as tumor necrosis factor, interleukin-1 (IL-1) alpha and beta, nuclear factor kappa B, mitogen-activated protein kinase, IL-22, and IL-17A [29,30]. Other studies also found a prominent role of the epidermal growth factor receptor (EGFR) in promoting the infection and serving as a target for antifungal drugs such as cetuximab or gefitinib [30].

### 4.2. M. lusitanicus, the Traditional Model for Genetic Manipulation in Mucorales

The traditional model organism in the study of *Mucorales* was the fungus *Phycomyces blakesleeanus*, promoted by Max Delbrück during the middle of the last century. However, the reluctance of *P. blakeskeeanus* to accept the modern genetic transformation technologies relegated it to classical genetic approaches. The lack of an efficient genetic transformation model among *Mucorales* delayed the study of these fungi compared to other fungal groups [82]. The genetic study of *Mucorales* was resumed with the first protocol to transform *M. lusitanicus* using self-replicative plasmids [83] (Table 2). This protocol is based on the formation of protoplasts by the digestion of the cell wall and the following introduction of plasmids molecules by polyethylene glycol (PEG). Years later, the efficiency of the protocol was improved by substituting the PEG step with the technique of electroporation [7].

The clue for the first transformation using self-replicative plasmids was the previous identification of auxotrophies and the genes to complement them. The first auxotrophic marker was leucine, with *leuA* being the corresponding complementing gene. Later, the auxotrophy for uracil with mutations in two genes (*pyrG* and *pyrF*) was also implemented [83,87,88]. Plasmids containing these selectable markers were used to harbor other genes trying to complement mutant strains, which happened most frequently but not in all the transformants (Table 2). A small proportion of the transformants showed a phenotype silencing the expression of both the endogenous gene and the one present in the plasmid [84,85]. These studies represented the first discovery of the RNA interference (RNAi) mechanism in *Mucorales*, which later was fully dissected [64,65,66,67,68,74,85,89] (Table 2). The availability of self-replicating plasmids and the characterization of the RNAi mechanism allowed the first functional genomic approach in *Mucorales* (described above).

#### Homologous Recombination and Related Genetic Tools in *M. lusitanicus*

The first transformation protocol using self-replicative plasmids was the foundation for the first homologous recombination event. The procedure substituted the plasmid for a construct containing the marker gene and homologous arms upstream and downstream of the targeted locus [84]. From then on, the homologous recombination allowed the development of the main related genetic tools for the edition of the *M. lusitanicus* genome (Table 2).

The first application of homologous recombination techniques in *Mucorales* was the generation of knockout mutants by gene disruption or gene deletion. This technology led to the first genetic dissection in *Mucorales*, specifically in the dissection of the carotenogenic pathway. Structural genes are *carB* and *carRP* (mutants showing an albino phenotype), and regulatory genes are *crgA* (mutant showing a carotene overproducing phenotype). Additionally, the white-collar family (*mcwc1a*, *mcwc1b*, and *mcwc1c:* mutants showing altered phototropism, *crgA*-dependent regulation, and light-defective induction; respectively) were functionally studied, proposing a complex model to explain the regulation of the light responses in *Mucorales* [84,90,91,92,93,94,95]. Later, the RNAi machinery of *M. lusitanicus* was thoroughly studied, dissecting all the steps of the canonical pathway: the triggering and amplification (genes *rdrp1* and *rdrp2*, mutants showing RNAi-defective phenotypes) [64,69], the production of siRNAs (genes *dicer1* and *dicer2*, mutants showing RNAi-defective phenotypes) [65,89], and the targeting machinery (genes *ago1*, *ago2*, and *ago3*, mutants showing RNAi-defective phenotypes)) [66]. These studies unveiled, for the first time in fungi, the role of the RNAi mechanism regulating endogenous genes involved in fungal physiology [67,96,97]. At the same time, the dissection of the canonical RNAi pathway led to the identification of the epimutational pathway and the NCRIP, described above.

Besides gene deletion, gene overexpression can be easily achieved by adding a target gene to the engineered construct with either its own promoter or a stronger one (*Pgpdh*) [25,70]. This strategy has been designed in *M. lusitanicus* to integrate the construct into the *carRP* locus, a gene involved in the synthesis of the colored compound beta-carotene, therefore generating albino transformants that are easily identified [98]. Finally, protein tagging has also been optimized in *M. lusitanicus* by replacing the original gene sequence with a fusion construct containing the in-frame tag. This methodology was recently implemented in *M. lusitanicus* by expressing fluorescent proteins that identified the centromeres and their particular structure [86].

### 4.3. Rhizopus Microsporus, the First High-Virulent Mucoral Available for Genetic Analysis in Mucorales

*Rhizopus* species are the most frequent isolates found in patients suffering mucormycosis, ahead of other genera such as *Mucor* or *Lichtheimia* [99]. *Rhizopus microsporus* presents two interesting features that make it a unique study model among mucoralean species. First, it is a highly virulent fungus that not only infects animals, producing mucormycosis, but also infects plants, causing rice seedling blight [100]. Secondly, *R. microsporus* cells may harbor endosymbionts, special bacteria living in the fungal cytoplasm in a symbiotic relationship with the fungus [101]. Endosymbiotic bacteria are related to plant infection through the production of rhizoxin, a toxin that blocks plant cell mitosis and necrotizes plant tissue, favoring the development of *R. microsporus* [100]. Regarding mucormycosis, these symbiotic bacteria drive phagocyte evasion and opportunistic virulence [102].

Although the features described above point to *R. microsporus* as the perfect model to study virulence and mucormycosis, its reluctance to genetic manipulation delegated these studies to the less virulent fungus *M. lusitanicus*. Fortunately, recent work has successfully developed a CRISPR-Cas9 plasmid-free methodology for the genetic manipulation of *R. microsporus* [9,103]. This work designed a platform for virulence studies in *R. microsporus* that included a specifically selected initial strain and positive and negative controls, besides the optimized methodology for successful homologous recombination.

Regarding the obtention of the initial strain, such as in *M. lusitanicus*, a uracil auxotrophic strain of the *R. microsporus* wild-type strain ATCC 11,559 was isolated, with the critical difference of selecting a spontaneous mutant without undergoing induced mutagenic methodologies. This strategy complicates the isolation of the auxotrophic mutant but ensures that the final strain has a clean background without unknown mutations that could interfere with the virulence assays when testing candidate genes. Once the uracil auxotrophic strain was characterized as harboring a non-synonymous substitution in the *pyrF* gene, this strain served to generate another auxotrophic strain for leucine. In this strategy, the wild-type *pyrF* gene was used to disrupt the gene *leuA*, rescuing the uracil auxotrophy. The virulence of all these strains was tested by optimizing the conditions for a simple murine model that consisted of Swiss mice without any immunosuppression treatment. The elimination of the immunosuppression treatment of the host represents another essential advantage of *R. microsporus* as a new study model. These virulence assays showed a prominent avirulent phenotype among the strains incapable of producing their own uracil and a full restoration of virulence in those complemented with a functional *pyrF* gene. Thus, the platform for virulence assays using *R. microsporus* offers single and double auxotrophic strains for the construction of single or double mutants; wild-type and *LeuA^-^* strains as positive controls showing a full virulence potential; and *pyrF^-^* strains as negative controls showing an avirulent phenotype (Table 2).

The methodology to successfully transform *R. microsporus* involves the digestion of the cell wall, similar to the transformation of *M. lusitanicus*. However, the digestion of the cell wall of *R. microsporus* requires a higher concentration of lytic enzymes and adjusted temperatures and times. After the digestion, the introduction of the recombinant DNA also requires the optimization of the electroporation parameters [9,104]. The recombinant DNA represents another advantage with respect to the previous protocol used in *M. lusitanicus* because it only requires two flanking micro-homology regions (35–40 nt) for effective homologous recombination. Along with this simple recombinant DNA, the electroporation introduces ribonucleoproteins containing Cas9 and a gRNA into the cells, which is designed to cut homologous sequences to the flanking regions of the recombinant DNA. Following this methodology, the authors generated the first mutant strain and showed the first visual phenotype in *Rhizopus*. Specifically, a mutant in the gene *crgA* produced a phenotype with defective aerial mycelia and spores containing higher amounts of melanin, although this mutant did not show alterations in virulence [9].

## 5. Conclusions

**Figure 1:**
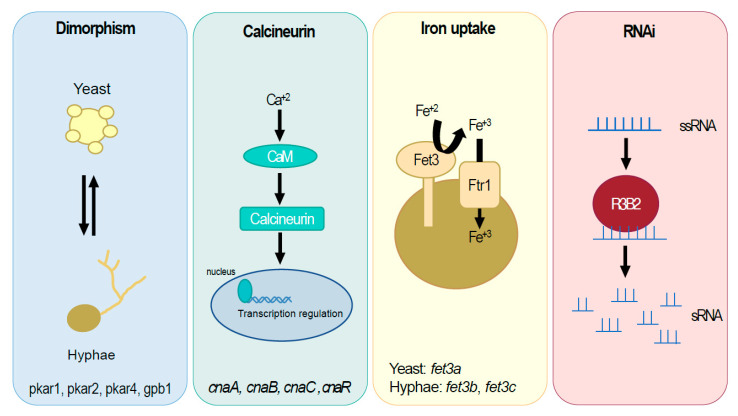
Illustration showing the main pathways and genes that are related to virulence in *Mucorales*.

In this review, we have compiled all the advancements in the genetic manipulation of *Mucorales* that have contributed to the understanding of mucoralean molecular biology, with a specific emphasis on examining mucormycosis (Figure 1). Many of these studies focused on virulence factors previously identified in other pathogens as a logical approach [105]. Thus, one of the most studied targets is the iron-uptake systems of *Mucorales*, as iron is a crucial component for most organisms and has previously been identified as a virulence factor in other pathogens. These new studies have uncovered a correlation between the iron-uptake system and dimorphism, suggesting the presence of unknown regulatory factors that could be targeted for new treatments [15]. Besides its connections with the iron-uptake system, the very mechanism of dimorphism is directly related to virulence, offering its fungal-specific machinery as a promising target of new antifungal compounds [26,27]. Additionally, research on dimorphism and gene silencing has led to the discovery of a novel mechanism of antifungal resistance through the epigenetic silencing of target genes [10]. Unfortunately, the RNAi mechanism is widely found in eukaryotic organisms, lacking effectiveness as a specific target of antifungal compounds. Nevertheless, a deeper study of the epimutational pathway found that it is regulated by non-canonical RNAi machinery, which mainly depends on the unconventional RNase III R3B2 [30,69,71]. This special RNase III can be proposed as a promising target for new antifungal compounds as it is uniquely found in *Mucorales*. Another promising avenue of research is the use of specific antibodies against conserved regions of CotH proteins, which has shown good results in animal models.

Although the study of virulence factors commonly found in different pathogens might represent a reasonable approach, the unique virulence and antifungal resistance of *Mucorales* suggests a complex interplay of factors that are specific to this group of fungi [106]. In this sense, the dissection of mucoralean pathogenic potential must also include approaches designed to find new virulence factors undescribed in other pathogens. Whole-genome sequencing of several *Mucorales* species has revealed a significant number of “unknown function genes” that may play a critical role in their virulence [103,107]. The study of an unknown function gene in *Mucorales* represents a challenge with more complexity than a gene with information previously obtained in other organisms. However, if we want to address the unusual and unique behavior of mucoralean pathogens, we will also have to face these challenges in future approaches.

Regarding the study models, the wide range of genetic tools now available in *M. lusitanicus* demonstrates that this fungus remains the most suitable study model for the genetic dissection of various cellular processes in *Mucorales* [108,109]. The current possibilities to manipulate *M. lusitanicus* include gene deletion, interruption, complementation, overexpression, labeling with fluorescent makers, amino acid substitution, RNAi silencing, and functional genomic libraries [110,111]. More recently, *M. lusitanicus* has served as a new model to study adenine methylation in eukaryotes [112]. Therefore, regarding the pathogenesis of *Mucorales*, *M. lusitanicus* has been the primary genetic model of the past decade. However, it is worth noting that *M. lusitanicus* has shown reduced virulence in laboratory survival assays using murine models and has never been isolated as a causing agent of mucormycosis. The recent development of methodologies allowing stable homologous recombination in *R. microsporus*, one of the most common causing agents of mucormycosis, represents a significant breakthrough in the study of mucormycosis [9,103]. This development will make *R. microsporus* the preferred choice for future studies related to virulence.

## Figures and Tables

**Table 1:** Genes recently involved in the virulence potential of *Mucorales*.

Study Model	Gene	Function	Reference
*M. lusitanicus*	*fet3a*	Iron uptake	[15]
*M. lusitanicus*	*fet3b*	Iron uptake	[15]
*M. lusitanicus*	*fet3c*	Iron uptake	[15]
*R. delemar*	*ftr1*	Iron uptake	[16]
Several	*cyp51 f1*	Ergosterol synthesis	[19]
Several	*cyp51 f5*	Ergosterol synthesis	[19]
*M. lusitanicus* and *R. delemar*	*cotH* family	Cell wall antigen	[20,21]
*M. lusitanicus*	ID112092	Secreted, unknown	[22]
*M. lusitanicus*	*wex1*	Exonuclease, unknown	[23]
*M. lusitanicus*	*atf1* and *atf2*	Transcription factors	[24]
*M. lusitanicus*	*mcplD*	Signaling	[25]
*M. lusitanicus*	*mcmyo5*	Intracellular transport	[25]
*M. lusitanicus*	*cnaA*, *cnaB*, *cnaC*, and *cnaR*	Calcineurin, pleiotropic	[26,27]
*M. lusitanicus*	*pkar1*, *pkar2*, and *pkar4*	Dimorphism	[28]
*M. lusitanicus*	*gpb1*	Dimorphism	[29]
*M. lusitanicus*	*r3b2*	RNAi	[30]
*Rhizopus microsporus*	*pyrF*	Uracile synthesis	[9]

**Table 2:** Main genetic tools in *Mucorales*.

Study Model	Genetic Tool	Year of Development	Reference
*M. lusitanicus*	Plasmid transformation	1984	[7,83]
	Stable homologous recombination	2001	[84]
	Gene complementation	2001	[84]
	RNAi	2003	[85]
	Amino acid substitution	2015	[70]
	Genomic RNAi libraries	2017	[70]
	Fluorescent protein tagging	2019	[86]
*R. delemar*	Plasmid transformation	2010	[16]
	RNAi	2010	[16]
	Unstable homologous recombination (Heterokaryons)	2010	[16]
*R. microsporus*	Plasmid transformation	2021	[9]
	Stable homologous recombination	2021	[9]
	Gene complementation	2021	[9]

## Data Availability

Not applicable.
